# The impact of coal trains on PM_2.5_ in the San Francisco Bay area

**DOI:** 10.1007/s11869-023-01333-0

**Published:** 2023-03-15

**Authors:** Bart Ostro, Nicholas Spada, Heather Kuiper

**Affiliations:** 1 Air Quality Research Center, University of California, Davis, CA USA; 2 Oakland, CA USA

**Keywords:** Particulate matter, PM_2.5_, Train, Rail, Coal, Health

## Abstract

Exposure to fine particulate matter (PM_2.5_) is associated with adverse health effects, including mortality, even at low concentrations. Rail conveyance of coal, accounting for one-third of American rail freight tonnage, is a source of PM_2.5_. However, there are limited studies of its contribution to PM_2.5_, especially in urban settings where residents experience higher exposure and vulnerability to air pollution. We developed a novel artificial intelligence-driven monitoring system to quantify average and maximum PM_2.5_ concentrations of full and empty (unloaded) coal trains compared to freight and passenger trains. The monitor was close to the train tracks in Richmond, California, a city with a racially diverse population of 115,000 and high rates of asthma and heart disease. We used multiple linear regression models controlling for diurnal patterns and meteorology. The results indicate coal trains add on average 8.32 µg/m^3^ (95% CI = 6.37, 10.28; p < 0.01) to ambient PM_2.5_, while sensitivity analysis produced midpoints ranging from 5 to 12 µg/m^3^. Coal trains contributed 2 to 3 µg/m^3^ more of PM_2.5_ than freight trains, and 7 µg/m^3^ more under calm wind conditions, suggesting our study underestimates emissions and subsequent concentrations of coal train dust. Empty coal cars tended to add 2 µg/m^3^. Regarding peak concentrations of PM_2.5_, our models suggest an increase of 17.4 µg/m^3^ (95% CI = 6.2, 28.5; p < 0.01) from coal trains, about 3 µg/m^3^ more than freight trains. Given rail shipment of coal occurs globally, including in populous areas, it is likely to have adverse effects on health and environmental justice.

## Introduction

Myriad large epidemiological studies definitively establish that exposure to fine particulate matter (particles less than 2.5 microns in diameter or PM_2.5_) is associated with a wide range of adverse health effects. Exposure to PM_2.5_ has been linked to premature mortality, cardiovascular, cerebrovascular, and respiratory diseases, other chronic diseases, adverse birth outcomes, and cognitive and developmental impairments (WHO 2021, U.S. EPA 2019). These effects occur even at concentrations lower than current regulatory standards (Brunekreef et al. 2021).While most studies of PM2.5 have examined daily or multi-year exposures, there is evidence of health effects from exposures of as short as one hour (Liu et al. 2021; Peters et al. 2001; Wu et al. 2020). The World Health Organization recently lowered their air quality guidelines and indicated there is no known safe level of PM_2.5_ (U.S. EPA 2019; WHO 2021). The recent study of the Global Burden of Disease estimates that exposure to PM_2.5_ contributed to 6.7 million deaths per year worldwide, nearly 12% of the global total and the fourth highest risk factor for global mortality (Fuller et al. 2022). Of note, exposure to PM_2.5_ constitutes an environmental justice concern as exposure and adverse effects are borne disproportionately by the most vulnerable, including infants, children, the elderly, people of color, those with low incomes, and those with underlying health conditions (Tessum et al. 2021).

Recent studies report that the combustion of fossil fuels, including coal, oil, and natural gas, is the largest source of ambient PM_2.5_-related mortality with coal the largest source of this mortality (Vohra et al. 2021; McDuffie et al. 2015). Combustion, however, is not the only source of coal-related particulate matter as fugitive dust from rail transport is known to be significant (BNSF Railway 2011). Trains transport nearly 70% of coal deliveries in the United States, with coal accounting for 1 of every 3 tons of American rail freight (US Energy Information Administration 2022). In a note to its customers, the BNSF Railway’s own assessment stated: “The amount of coal dust that escapes from PRB [Powder River Basin in Wyoming and Montana] is surprisingly large” and reports have indicated that as much as 3% of the coal loaded into a coal car can be lost in transit (Baruya 2012; BNSF Railway 2011). Studies have confirmed that coal trains produce particulate matter through not only engine diesel emissions but also directly from the coal. These latter emissions are via blow-off, suspension, and re-entrainment from wind erosion and wind scouring of loaded and unloaded coal cars, door leakage, and the “parasitic load, i.e., coal spilled and carried on external parts of the train (Prakash et al. 2018). The magnitude of ambient particulates from coal trains are influenced by train and wind speed, weather, moisture, rail car and load geometry, physical properties of the coal, vibration, and the use and efficacy of dust suppression methods (Prakash et al. 2018). Unfortunately, the actual contribution of coal trains to ambient PM_2.5_ is poorly documented.

Given the dearth of studies quantifying the effects of coal transport on subsequent concentrations of particulate matter and the significant health implications of exposure to particulate matter, additional study is warranted. Below, we report results from the novel monitoring system we developed and utilized to quantify the contribution to ambient PM_2.5_ from uncovered railcars that convey coal predominantly from mines in Southern Utah to the Levin Terminal in Richmond, California.

## Methods and materials

### Data collection

Particulate matter from coal is known to contain many impurities and elements including heavy metals known to be toxic or carcinogenic to humans (OEHHA 2015). Specifically, the coal of interest in this study originated from the Wasatch Plateau coal fields, a coal-bearing outcrop approximately 145 km long and 11 to 32 km wide (Hatch et al. 1979). Previous assessments have determined coal from the plateau to be high volatile bituminous (Hatch et al. 1979). The coal is primarily carbonaceous with various inclusions and impurities, including several mineral species along with elemental impurities of Cr, Ni, and Se. There are also trace elements including As, Ba, Cd, F, Mn, Sb, Sr, Th, U, and V (Hatch et al. 1979).

**Fig. 1:**
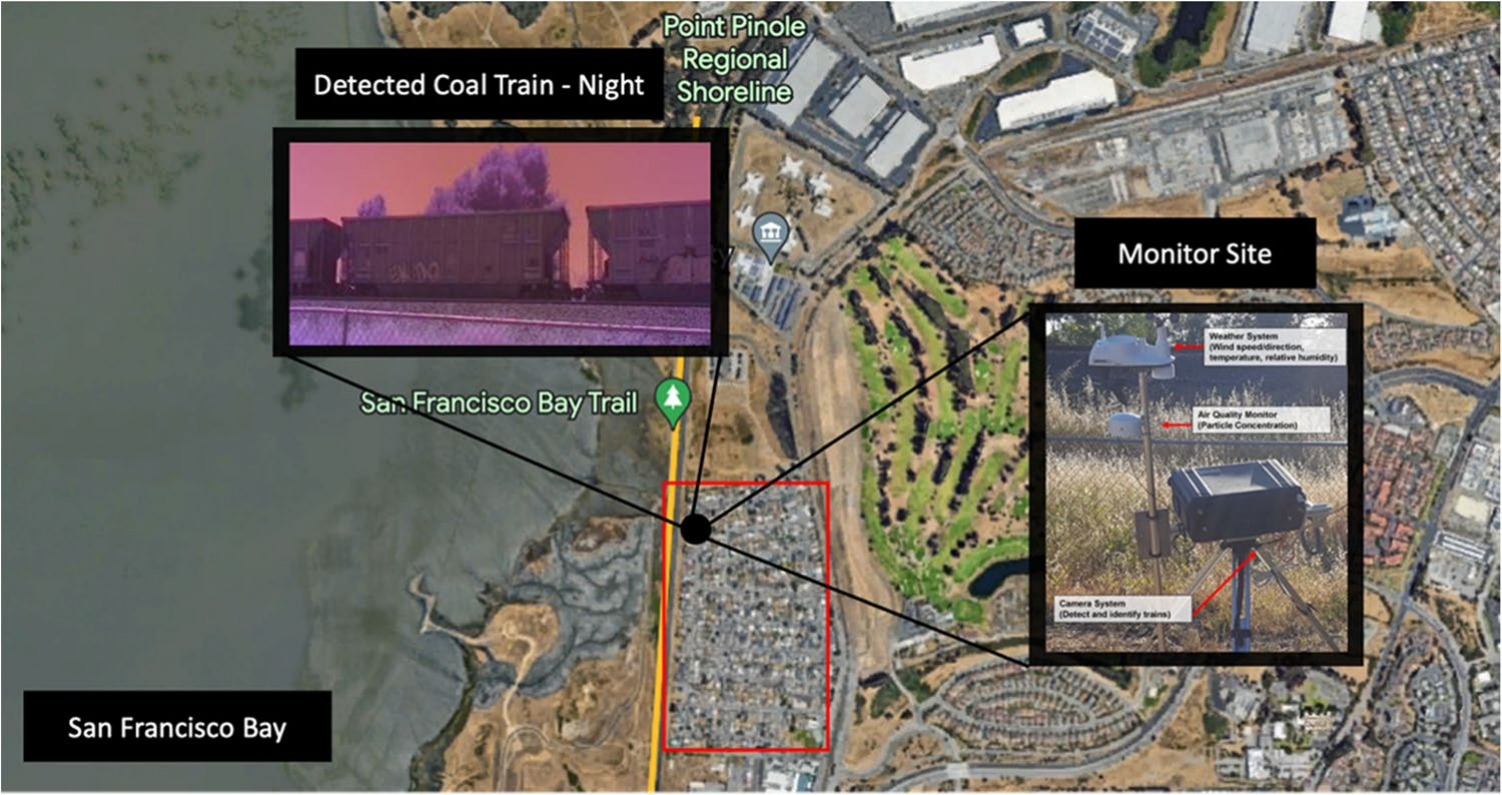
Location of monitor site and surroundings

To determine the PM_2.5_ concentration resulting from passing full and unloaded (“empty”) coal, freight and passenger trains, passing trains were monitored from May 19, 2022 through October 31, 2022 at a populated residential site approximately 7 km north of the terminal. The site is near the culmination of an 800-mile journey, thereby capturing the realistic conditions of long-haul coal conveyance as compared to the conditions at departure where dust suppressants are freshly applied, and trains are optimally loaded. The monitoring site is approximately 21.5 meters east (generally downwind) of the rail line, with parkland to the east and the San Francisco Bay to the west (Fig. 1). The site was selected to avoid PM_2.5_ from other important sources such as major roadways, industrial facilities, Richmond port operations and the Levin terminal itself. This location and our study methodology ensured that any observed changes in PM_2.5_ as the trains passed were strictly due to the trains themselves.

The train monitoring system comprises three data collection systems:

1. A personal weather station
2. An air quality sensor
3. A custom camera system

The personal weather station was selected for direct data output via serial communication (VantageVue, Davis Instruments, USA). It provides temperature, ambient pressure, relative humidity, precipitation, and other meteorological parameters. The meteorological data is collected every one-minute. In addition, hourly wind speed and direction were derived from the NOAA site in Richmond for comparison.

The air quality sensor is a custom package consisting of three optical PM sensors (PMS5003, Nanchang Panteng Technology Co., Ltd, China). These are equivalent to cell-reciprocal nephelometers and are commonly recognized as the sensors used in the widely-distributed PurpleAir PA-II monitor (Ouimette et al. 2022). The sensor responds to optical scattering from a 657 nm laser. Therefore, it is associated with mass via the mass scattering coefficient, which is a function of the chemical, morphological, and optical properties of the observed particles. The accuracy of this determination is governed by the variability of particle characteristics in the temporal and spatial dimensions. The sensors’ high temporal resolution of one second and their inter-instrument precision, as assessed by numerous field and laboratory studies, were the principal qualities that enabled the detection of rapid train events (Tsai et al. 2020; AQ-SPEC 2022). Three channels were included to strengthen data quality control and calculate variance for each observation. The raw data from all three sensors was collected every second.

Data quality metrics of the PM2.5 data were evaluated for 1 s, 10 s, and 10 min, equivalent to instantaneous readings, train event averaging, and pre-event background conditions, respectively. Prior to evaluation, the data was cleaned to remove aberrant sensor readings. Specifically, values outside two standard deviations were omitted. In all cases, these values were excessively high readings from the low-cost sensor. The observations used in the subsequent statistical analysis ranged from 0 to 117.45 µg m^−3^ with a median uncertainty of 27%, well within the linearity range of the sensors of < 300 µg^3^ (Barkjohn et al. 2022).

The custom camera system consists of a microcomputer (Jetson Nano, Nvidia, USA), a camera (NoIR PiCamera, Raspberry Pi), an artificial intelligence (AI) accelerator (Coral Edge TPU, Google, USA), a solid-state hard drive (500 GB T5, Samsung, S. Korea), and an infrared floodlight (IR Illuminator 30 deg, Axton Technologies, USA). The system is placed approximately 60 m from the chosen source and operates autonomously on a continuous basis, except for a daily 30-min period when data is being uploaded to a cloud server (Lightsail, Amazon Web Services, USA).

**Fig. 2:**
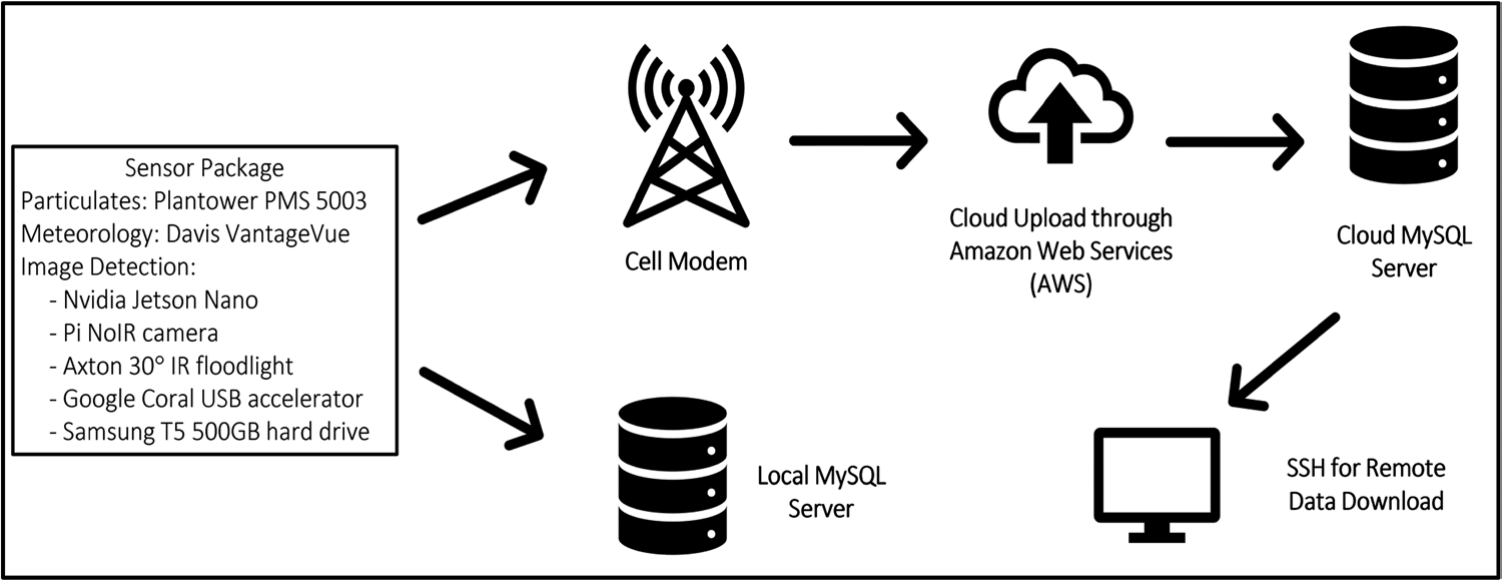
A schemata of the data collection system

The camera system is the pivotal technology that enables detection of passing trains. Images from the camera are passed to the computer at 30 frames per second, where they are pre-processed and passed to the AI accelerator. The accelerator is a Tensor Processing Unit (TPU, Coral Edge TPU, Alphabet, USA), which runs an image classification model customized for the monitoring location. This model identifies whether or not a train is present in the image. If so, the computer creates a train event and records: one second before the train was detected, the entire train event, and one second after the train is no longer detected. This recording is saved as an individual train event to an external hard drive. Train speed (meters per second) and the train direction towards or away from the terminal were determined during manual post-processing of the data. Determining object velocity from video recordings is error prone due to variable image processing rates. Instead, train speeds were estimated by using the average frame rate (frames per second) recorded during the monitoring period and fixed observation points in the camera’s field of view. A schematic diagram of the system is presented in Fig. 2.

For each 24-h period, data was aggregated from all three data sources and standardized into one second observations for each measurement parameter including meteorology, PM_2.5_ concentrations and train detection. During this < 30-min period, the monitoring systems were disabled and the data file along with associated video files were uploaded to the cloud server. The data aggregation and upload period were scheduled in the early morning hours when train activity was determined to be consistently absent. Files located in the cloud were retrieved at the user’s convenience for post-processing, which consisted of associating particulate matter and meteorological data with the observed train events based on the shared data timestamps. Accurate date and time determinations were ensured by consistent internet connection and verification by the operating system. Further detail on the derivation of the variables used in our analysis is provided in Appendix A.

### Data management

The PM_2.5_ during train passage was recorded in one-second concentrations and averaged for the roughly 4 to 5 min of passage (longer for freight trains). In addition to the PM_2.5_ average during the passage of the train, the maximum 10-s average concentrations during the train passage were also recorded and analyzed in order to compare with previous studies.

To determine the change in PM_2.5_ due to passing trains, we quantified the difference between the measured PM_2.5_ at the rail site and a “control” period of exposure. The control, also considered a “pre-exposure” period, corresponded to the period just prior to a train’s passage, allowing capture of ambient PM_2.5_ without the train’s contribution as well as controlling for normal diurnal and regional changes in PM_2.5_ concentrations. A generally similar approach was used by previous studies (Jaffe et al. 2015; Akaoka et al. 2017). We also established a gap between the control period and the train passage to ensure that particles influenced by the high-pressure zone in front of an oncoming train would not be included in the control.

To select the duration of the control exposure and this gap immediately before the train passage, we examined several alternatives including: a five-minute average ending with a 2- minute gap before the train (5/2) as well as 3/2, 5/5, 10/2, 10/5 and 10/10. Ultimately, the results were insensitive to the alternative control and gap periods, so only the results using 10/10 are reported below.

### Analysis

We addressed several issues including whether full or empty coal cars contribute to local ambient PM_2.5_ concentrations and, if so, by how much. We also compared the impacts of coal cars relative to those of both freight and passenger trains. Using multiple linear regression with the change in PM_2.5_ concentration as the dependent variable, our model included binary variables for each of the four train types (passenger, freight, empty and full coal) and examined and controlled for potential confounders. For example, a previous study found a strong association between PM_2.5_ from coal trains and the effective wind speed (the sum of train and wind speed) (Jaffe et al. 2015). To test the sensitivity of our results to the model specification, we examined the impact of several covariates including train speed, wind speed, effective wind speed, duration of exposure (based on the elapsed time of the train passing), average temperature, dewpoint and relative humidity. The inclusion of humidity served to control for the potential impact of the hygroscopic property of fine particles when measured with optical sensors. We ran the model without a constant term, which facilitated the direct comparison of the impact among the train types. The model results were identical to a model that adds a constant term and drops one of the train types to avoid multi-collinearity.

Additional sensitivity analysis included examining the impact of converting the negative values for the change in PM_2.5_ into zero values. The negative change from the control period could be a result of significant dust from activities at the monitor’s residential location, dust from trains occurring in the control period, or from a sudden change in wind speed or direction prior to the train arrival. We also considered subsets of certain covariates. For example, we examined those days where wind was below the mean level of 3.1 mph since these calmer periods may relate to higher concentration at the nearby monitor, whereas particles may disperse to a larger area under other wind conditions. Finally, we tested a model where the air quality sensor was calibrated and directly corrected for relative humidity using the closest Federal Equivalency Method (FEM) monitor to our site. This monitor was located in nearby San Pablo (Air Quality System Site ID: 06–013-1004), 1.6 km from our site and generated the following fit, with an R^2^ of 0.58:

$$\mathrm{PM}2.5\_\mathrm{C}=9.79+.76*PM\_PA-0.095*humidity$$

where PM2.5_C is the calibrated and corrected concentration of PM_2.5_, and PM_PA is the original reading at the train site. In addition to the average change in PM_2.5_ (difference of PM_2.5_ during train passage and the control), the maximum (10 s average) concentration relative to the control period was analyzed to compare with findings of previous studies.

## Results

Ultimately, during the six-month observation period, the increases in ambient PM_2.5_ concentrations were measured during the passage of four different train types. Complete data were available for full coal trains (n = 15), empty coal trains (n = 14), freight trains (n = 568) and passenger trains (n = 2235) as identified by the video recordings from the camera system described above. There were some significant differences between characteristics of the train types (see Appendix B for detailed summary statistics). For example, focusing on freight trains versus full coal trains, the mean duration (in seconds) and speed (m/s) for the former were 236 and 18.3, versus 144 and 12.5 for the latter. At the other extreme, the means of these same parameters for passenger trains were 2.2 s and 31.7 m/s.

The results for the basic regression model are presented in Table 1. As expected, wind and train speed were both statistically significant. In addition, the passage of an empty coal car contributed about 2.3 µg/m^3^ (95% CI = -0.28, 4.82; p < 0.1) to the ambient air, while freight and full coal cars contributed 4.5 µg/m^3^ (95% CI = 3.82, 5.18; p < 0.01) and 6.8 µg/m^3^ (95% CI = 4.34, 9.24; p < 0.01). Controlling for the direction of the freight train did not alter the results. This finding indicated that the regression coefficients of these three train types (freight and full/empty coal) were statistically significant from zero and also statistically different from each other. In contrast, the PM_2.5_ increment from passenger trains was relatively small and not significantly different from zero, so it was not included in the sensitivity analyses below. The amount of explained variation from the basic model was relatively low at 16%.

**Table 1:** Regression Results for Basic Model

Variables	PM_2.5_ave	PM_2.5_max
Windspeed	-0.256***	-3.865***
	(0.0628)	(0.742)
Train speed	0.0131**	0.185*
	(0.00582)	(0.089)
Passenger	0.257	7.785
	(0.455)	(7.265)
Empty Coal	2.274*	12.179
	(1.301)	(8.194)
Freight	4.496***	18.498***
	(0.347)	(4.095)
Full Coal	6.794***	22.838***
	(1.252)	(7.484)
R^2^	0.16	0.25
n	2829	550

PM_2.5_ave = average during train passage; PM_2.5_max = maximum 10 s during train passage; standard errors in parentheses

^***^ p < 0.01, ** p < 0.05, * p < 0.1

The findings of the sensitivity analysis are displayed in Table 2. Given the null impact of passenger trains, further results for this mode were not included. Model (1) reproduces the results of the basic model. Model (2) added the average temperature during the one-hour average that included the train passage, and resulted in increases in the PM_2.5_ impact for all three train types with empty coal, freight and full coal cars contributing 5.6 µg/m^3^ (95% CI = 2.5, 8.7), 7.5 µg/m^3^ (95% CI = 5.8. 9.2) and 9.7 µg/m^3^ (95% CI = 6.8, 12.6), respectively. All were statistically significant with p < 0.01. Model (3) indicates the impact of adding humidity which resulted in reductions of approximately 2.5 µg/m^3^ from the basic case. Model (4) again includes humidity but assigns a zero value when the change in PM_2.5_ was negative. This adjustment slightly increased the PM_2.5_ contribution of all of the train types. Model (5) adds a control for dewpoint, a combination of temperature and humidity, which resulted in an increase in the change in PM_2.5_ from the basic model, while in Model (6) observations are restricted to those occurring during calm wind conditions (less than the mean of 3.0 mph). This constraint significantly increased the contribution of coal trains to ambient PM_2.5_ to 12.1 µg/m^3^ (95% CI = 7.7, 16.5; p < 0.01) versus 5.1 µg/m^3^ (95% CI = 3.8, 6.4; p < 0.01) for freight cars. Finally, Model (7) uses the data from the calibrated PM_2.5_ concentrations and generated statistically significant estimates of 8.3 µg/m^3^ (95% CI = 6.4, 10.3; p < 0.01) and 6.5 µg/m^3^ (95% CI = 6.0, 7.1; p < 0.01), respectively, for full coal and freight trains. Models (1) through (6) each exhibited modest R^2^ less than 0.19. However, the calibrated Model (7), which provided a robust correction for humidity, explained 53% of the variation in the change in PM_2.5_. Additional model specifications of Model (7) with covariates used in the earlier models such as train duration, effective wind speed or quadratic terms failed to improve the model fit.

**Table 2:** Regression Results of the Increase in Average PM_2.5_ (µg/m^3^) in Alternative Models

Train type	1	2	3	4	5	6	7
Empty Coal	2.27*	5.64***	0.96	1.10	5.84***	2.19	6.41***
Freight	4.50***	7.49***	2.78***	3.05***	7.80***	5.07***	6.53***
Full Coal	6.79***	9.71***	5.09***	5.20***	10.00***	12.12***	8.32***
R^2^	0.16	0.16	0.16	0.18	0.16	0.20	0.53
n	2829	2829	2829	2829	2829	1330	2829

^***^ p < 0.01, ** p < 0.05, * p < 0.1

Models are: (1) Basic (2) Basic + temperature (3) Basic + humidity 4) Basic + humidity + negatives = 0 (5) Basic + dew point (6) wind speed < mean (7) Calibrated monitor. (Note: Basic model includes wind speed, train speed and a binary variable for each train type, no constant)

Table 3 displays the regression results for the increase in peak (10 s average) PM_2.5_ concentrations above the control concentrations during the passing of full coal cars (n = 18), empty coal cars (n = 16) and freight cars (n = 653). Results for passenger trains were not included since few of these trains had durations that were 10 seconds or more. The model specifications were similar to those used in the previous analyses and included wind speed, train speed and the 3 train types. Given the above findings, we focused on 3 different models: a basic model (Model 1), a model corrected and calibrated for humidity as above (Model 2), and the calibrated model under calm wind conditions defined as average wind less than the mean (Model 3).

**Table 3:** Regression Results of the Increase in Peak PM_2.5_ (µg/m^3^) in Alternative Models

Train type	1	2	3
Empty Coal	12.8	9.26	8.09
Freight	18.50***	14.06***	14.37***
Full Coal	22.84***	17.37***	19.96 **
R^2^	0.25	0.25	0.27
n	550	550	360

^***^ p < 0.01, ** p < 0.05, * p < 0.1;

Models are: (1) Basic (2) Calibrated monitor (3) Calibrated monitor with wind < mean

Basic model includes wind speed, train speed and a binary variable for each train type

For the basic model, the results indicated an increment in maximum PM_2.5_ over the control period of 22.9 µg/m^3^ (95% CI = 8.1, 37.5); p < 0.01) for full coal trains. For the model calibrated and corrected for humidity, the increment from coal cars was 17 µg/m^3^ (95% CI = 6.2, 28.5; p < 0.01) while the corresponding change in PM_2.5_ was 14.1 µg/m^3^ (95% CI = 7.9, 20.2; p < 0.01) for freight trains and 9.3 µg/m^3^ (95% CI = -3.0, 21.5, NS) for empty coal cars. Under calm wind conditions, the impact from coal cars increased to almost 20 µg/m^3^ (95% CI = 3.4, 36.6; p < 0.05) while the freight increment did not change from the previous case.

## Discussion

Our results indicate that the average change from passing coal trains adds approximately 8.32 µg/m^3^ (95% CI = 6.37, 10.28; p < 0.01) to the ambient PM_2.5_, with a range of midpoint estimates, based on the sensitivity analysis, of 5 to 12 µg/m^3^. These results also suggest that full coal cars contribute approximately to 2 to 3 μg/m^3^ of PM_2.5_ more than freight trains observed in our Richmond, California sample. Strikingly, with very calm winds, the nearby concentrations from coal trains were about 12 μg/m^3^ versus 5.1 for freight trains. This suggests the possibility of our study underestimating the emissions and overall impact of dust from coal trains, since on windier days the dust may simply be dispersed over a wider region beyond our monitoring site. We also observed that unloaded coal cars tended to add 2 μg/m^3^ of PM_2.5_ to the existing ambient concentrations, with a range from our sensitivity analysis of from about one (non-significant) to over 5 µg/m^3^. Regarding peak (10 s) concentrations of PM_2.5_, the calibrated model indicated an increase of 17.4 µg/m^3^ (95% CI = 6.2, 28.5) from coal trains which tended to contribute about 3.5 µg/m^3^ more than freight trains across the models examined. Calm wind conditions resulted in an increase from coal trains of 20 µg/m^3^ (95% CI = 3.4, 36.6; p < 0.01).

Given the known bias of humidity on optical PM monitors, in addition to controlling for humidity and dewpoint directly in the model specification, a regression model was estimated using data calibrated and corrected for humidity using a nearby FEM monitor (Barkjohn et al. 2021). It is well established that mass calibrations of optical sensors are temporally and spatially dependent on particle optical characteristics (Dubovik et al. 2002; Bond and Bergstrom 2006). The assumption here is that consistent calibration factors from monitors within the same geographic region and time period are reasonable surrogates for in situ calibration.

There are only a few previous studies that have measured PM_2.5_ concentrations from coal trains. One study examined coal and freight trains passing through the rural Columbia River Gorge (Washington) in the summer of 2014 (Jaffe et al. 2015). The study examined the difference between the 10 s maximum PM_2.5_ and the background concentration. The authors observed a doubling in peak concentration for coal trains (20.9 µg/m^3^) versus freight trains (10.7 µg/m^3^). This is consistent with our results for coal trains using a similar averaging time of 17.4 µg/m^3^. The average effective wind speeds in the Jaffe study were much higher than those in our study and were often associated with very high concentrations of PM_2.5_. This suggests that PM_2.5_ concentrations associated with train passage are likely to be even greater in certain areas farther away from the City of Richmond’s urban setting due to greater train speeds.

A previous study collected data on coal trains operating in the Fraser River Delta area of British Columbia, Canada. In comparing ambient air impacts of the coal trains (n = 20) to background concentrations, the results suggested an increase of 5.3 (a 54% increase over background), 4.1, and 2.6 µg/m^3^, respectively, for PM_3_ (comparable to PM_2.5_), PM_10_, and PM_20_, with occasional spikes in PM_3_ from coal trains to 100 µg/m^3^ (Akaoka et al. 2017).

Another study collected data on a single day from four monitors located at varied distances from the train line on full (n = 10) and empty (n =11) coal trains heading to and from the Port of Newcastle in New South Wales, Australia (Higginbotham et al. 2013). For full coal cars, there were increases of 2.9 and 7.2 µg/m3, respectively for PM_2.5_ and PM_10_ and 7.1 and 18.9 for empty coal cars. Higher impacts for empty coal cars were also reported in studies by Katestone Environmental Pty Ltd (2013).

Finally, Ryan and Wand (2014) analyzed the impacts of freight, empty coal and full coal trains in the Hunter Valley in New South Wales, Australia (Ryan and Wand 2014). The crude (unadjusted) increases in PM_2.5_ for passing freight, empty coal and full coal cars was 0.53, 1.13 and 1.20 µg/m^3^, respectively; all statistically significant differences from baseline levels. Their measurements indicated that particulate level concentrations were elevated not only during but also prior to and especially after a train’s passing.

Most of the dust from coal trains occurs from the rail car (80%), with spilled coal (9%) and door leakage (6%) being other sources (Connell Hatch 2008). A consequence is coal dust deposition, with studies finding that, on average, coal composed 6—25% of deposited dust in rail corridors, although Akaoka et al. reports up to 90% in local dust (Akaoka et al. 2017; DSITIA 2015). Evidence indicates that particulate matter from coal trains, storage and open mines can disperse at least 500 m from the source (Trivedi et al. 2009; Akaoka et al. 2017; Srivastava et al. 2021; Sahu and Pakra 2022).

To put our results into perspective, the current U.S.EPA 24-h and annual average standards are 12 and 25 μg/m^3^, respectively, while the World Health Organization guidelines for the same averaging times are 5 and 10 μg/m^3^ (U.S. EPA 2019; World Health Organization 2021). In addition, both U.S.EPA and WHO indicate that there is no threshold or safe level for ambient PM_2.5_. Therefore, a hypothetical three coal trains per week in an urban area could represent an important increase in PM_2.5_ to nearby residents. Incremental concentrations would subsequently increase the risk of a wide range of health effects including: premature mortality, cardiovascular and respiratory hospitalization or urgent care visits, increases in or exacerbation of asthma, adverse birth outcomes (e.g., low birth weight, prematurity, birth defects and neurodevelopment), possible neurological impacts in children and adults (autism, Alzheimer’s, Parkinson’s) as well as functional impacts such as days with respiratory symptoms, restricted activity, and work or school loss (WHO 2021). As noted above, even acute PM_2.5_ exposures as short as one hour (or a few hours) can increase the risk of adverse health outcomes, including: acute myocardial infarction, hospitalization and emergency department visits for cardiovascular and respiratory disease, ambulance calls and asthma exacerbation (Yorifuji et al. 2014; Kim et al. 2015; Chen et al. 2019).

Our study has several advantages including the development of an AI-based platform for precise identification of train types during the day or night; real time measurement of PM_2.5_ and meteorology; siting of a monitor with only the trains as a source of PM_2.5_; and the ability to produce data on train direction and speed. There were also some shortcomings in our study. There was only a small number of full and unloaded coal cars due to the reduction in economic activity during the COVID-19 pandemic and related supply chain issues. There was only a single monitor to measure the impact of passing trains. This was due to both logistical constraints pursuant to the COVID-19 pandemic and the difficulty in finding monitor host sites that were not impacted by other PM_2.5_ pollution sources in Richmond, a city transected by major highways, refineries, other heavy industry and a port. There is the possibility of exposure misclassification if some of the freight trains also included coal cars. The low R^2^ in some of the regression models could be due to several factors including the assignment of hourly wind, temperature and humidity to the 4–5 min of train passage and uncertainty in estimating train speed and length. There were also unmeasured factors such as train weight and number of engines. Finally, it is important to note that our analysis did not include measurements of either ultrafine (particles less than 0.1 micron) or coarse particles (PM10) which will always be generated from the passing trains. Since there is substantial evidence of adverse health effects from both of these particle sizes, the actual health risks posed by passing coal trains are clearly underestimated in this present study (Adar et al. 2014; Ostro et al. 2015).

Identifying the source of fugitive dust is important in part because the implications of exposure extend beyond individual and population health effects to matters of environmental and racial justice (Mikati et al. 2018). While coal dust can have far-ranging population exposures, the communities in relatively close proximity to the rail lines will be disproportionately exposed. These residents are more likely to be of lower-income or people of color (or both) and also more vulnerable to adverse health outcomes (Hricko et al. 2014; Jha and Muller 2017).

Finally, the impacts of the rail transport of coal are compounding because it involves traversing thousands of kilometers, meaning multiple environmental justice communities are impacted. Ecosystems such as rivers and coastlines also receive extended exposure as the rails often trace their contours. Further, the climate change implications of coal transport, storage and handling are significant, ultimately resulting in up to 16% of US carbon pollution (Meyer 2019).

## Conclusion

In this paper, we have reported evidence of significant increases in PM_2.5_ due to passing coal-carrying trains in Richmond, California. The observed increases were greater than those produced by freight trains and passenger trains. Unloaded coal cars also generated increases in PM_2.5_, but at lower concentrations than full coal cars. Quantifying the contribution of coal trains in urban air populations is important since vulnerable communities are typically found in close proximity to rail lines. In addition, inevitable dispersion of PM_2.5_ will increase population exposure over a much wider area. Since shipment of coal by train occurs throughout the world and for many urban areas, it represents a significant public health hazard. Finally, to overcome technical challenges that have historically been barriers to the study of coal trains, we developed an artificial intelligence-driven monitoring platform to detect and quantify air pollution from passing trains. These advancements will contribute to future studies of health effects from mobile sources.

## Data Availability

Reasonable requests for the datasets generated and/or analyzed during the current study are available by contacting Dr. Nicholas Spada at njspada@ucdavis.edu.

## Appendix A. Data field descriptions

The following discussion provides more background regarding the data fields, their units, and their calculation.

- TrainStart:
  - This field reports the date and time, to the second, that a train was observed by the customized artificial intelligence camera system
- TrainType:
  - This study classified the observed trains into four types:
    - Passenger – either Amtrak or CalTrain trains
    - Freight – Union Pacific trains that are carrying various products on rail cars but not identifiable coal-bearing cars
    - Coal – Union Pacific trains exclusively carrying coal hopper rail cars, either full or unloaded
- PreTrainPM#:
  - These fields represent the air quality levels prior to the arrival of a train. We explored various combinations of averaging times (1, 3, 5 and 10 min) and gap lengths between the PreTrainPM average and the observed train start time (1, 2, 5 and 10 min. For example, PreTrainPM10-10 utilizes a 10-min average PM_2.5_ that is a 10-min gap between the averaging period and the train event start. This field is presented in micrograms per cubic meter.
- AvgPM:
  - This field is the average PM_2.5_ concentration, in micrograms per cubic meter, observed during the time that a train was observed at the monitoring location. Note that each second of recorded data is the average of three separate PM sensors, and this value is the average of those averages.
- MaxPM:
  - This field is the maximum 10-s PM_2.5_ concentration, in micrograms per cubic meter, observed during the time that a train was observed at the monitoring location. Again, the data point is the average of three separate PM sensors.
- PMdelta_10_10:
  - These fields represent the difference between the PM_2.5_ concentrations observed during the train event and the pre-event levels. They are calculated as:
    $$XPM-Pretrain\_10\_10$$
    Where XPM is either the AvgPM or the MaxPM,
- Meters per second:
  This field is the estimated speed of the train using a custom video processing algorithm. The video is separated into individual images. Two positions (x-coordinates) are selected on either side of each image to act as positional triggers. The pixel values (in RGB) are collected from every pixel in each of these two lines. As the trains pass, we anticipate the pixel values to oscillate more than in the rest of the image; therefore, a y-coordinate is chosen with the maximum relative standard deviation out of all the y-coordinates in each line. Since each image is related to a distinct timestamp, we compare the first derivative of pixel intensity change in each optical band (R, G, and B) to find the temporal difference in peaks. Specifically, we are calculating the lag time between when the train is observed in each of the two locations. The distance between these two points is calculated from a conversion factor of pixels per meter, as determined by image analysis of Amtrak engines and train cars, whose dimensions are publicly available. Knowing the distance traveled (in meters) and the time difference (in seconds), we converted and calculated the speed in meters per second.
- Direction:
  This field indicates the direction the train was traveling in the observation. The determination of this parameter uses the same algorithm described for the previous parameter (m/s). The derivative peaks analyzed inform whether the train is moving to the right (northward and away from the terminal) or left (southward or towards the terminal), which result in binary values of 1 and 0, respectively.

## Appendix B

Table

**Table 4:** Descriptive statistics for regression variables

Stats	Pmave	PMave_C	PMmax10	PMmax10_C	Wind	TS	Humidity	Temp
Passenger Trains								
Mean	.36212	-.15727	9.7675	-.15437	1.37	30.759	70.741	66.291
SD	1.7242	1.5461	7.8401	1.5532	.621	7.1571	15.351	7.6733
Min	-18.84	-9.4019	2.5	-9.4019	0	3.576	20	51.3
Max	29.84	13.801	39.75	13.801	3.62	44.253	95	93.1
N	2235	2235	20	20	2235	2235	2235	2235
Freight Trains								
Mean	4.2347	1.3748	22.745	2.5612	1.25	17.064	76.505	63.841
SD	9.8485	5.2076	31.526	7.3161	.657	5.6849	15.177	8.2692
Min	-9.8	-7.057	0	-5.2539	0	1.341	20	49.5
Max	68.46	36.208	157.73	49.002	3.88	38.889	96	100
N	568	568	507	507	568	568	568	568
Full Coal Trains								
Mean	6.3867	2.5569	24.305	4.2791	1.35	12.779	75.867	62.14
SD	13.3	6.5004	42.809	9.5825	.724	5.5336	16.274	7.929
Min	-.42	-2.5676	1.7	-2.0174	.441	6.705	49	53.5
Max	41.43	19.443	162.4	33.68	2.73	29.055	94	76.6
N	15	15	15	15	15	15	15	15
Empty Coal Trains								
Mean	1.935	1.5673	12.286	1.8331	1.38	15.837	60.286	71.943
SD	4.8405	2.5167	18.95	2.5482	.582	3.192	15.544	6.9966
Min	-.57	-1.0231	1.35	-1.0231	.661	10.281	37	58.4
Max	18.54	9	74.3	9	2.73	20.115	84	85.9
N	14	14	14	14	14	14	14	14

*PMave* Change in average PM_2.5_ (µg/m^3^), *PMave_C* Change in average Corrected PM_2.5_ (µg/m^3^), *PMmax10* Change in peak PM_2.5_ (µg/m^3^), *PMmax10_C* Change in peak Corrected PM_2.5_ (µg/m^3^), *Wind* Average wind speed (m/s), *TS* Train speed (m/s), *Temp* Average Temp (F)

4

## References

[CR1] Adar SD, Filigrana PA, Clements N, Peel JL (2014). Ambient Coarse Particulate Matter and Human Health: A Systematic Review and Meta-Analysis. Curr Environ Health Rep.

[CR2] Akaoka K, McKendry I, Saxton J, Cottle PW (2017). Impact of coal-carrying trains on particulate matter concentrations in South Delta, British Columbia, Canada. Environ Pollut.

[CR3] AQ-SPEC (2022) http://www.aqmd.gov/aq-spec, last access: 6 December 2022

[CR4] Barkjohn KK, Holder AL, Frederick SG, Clements AL (2022) Correction and Accuracy of PurpleAir PM2.5 Measurements for Extreme Wildfire Smoke. Sensors 22, no. 24 (January 2022):9669 10.3390/s22249669 10.3390/s22249669 PMC9784900 36560038

[CR5] Barkjohn KK, Gantt, B, Clements AL (2021) Development and Application of a United States wide correction for PM2.5 data collected with the PurpleAir sensor. Atmos Meas Tech 4(6) 10.5194/amt-14-4617-2021 10.5194/amt-14-4617-2021 PMC8422884 34504625

[CR6] Baruya P (2012) Losses in the coal supply chain. IEA Clean Coal Centre. https://usea.org/sites/default/files/122012_Losses%20in%20the%20coal%20supply%20chain_ccc212.pdf. Accessed 13 Oct 2022

[CR7] BNSF Railway (2011) Coal dust frequently asked questions [2011 version]. http://www.bnsf.com/customers/what-can-iship/coal/coal-dust.html

[CR8] Bond TC, Bergstrom RW (2006). Light Absorption by Carbonaceous Particles: An Investigative Review. Aerosol Science and Technology.

[CR9] Brunekreef B, Strak M, Chen J et al (2021) Mortality and Morbidity Effects of Long-Term Exposure to Low-Level PM2.5, BC, NO2, and O3: An Analysis of European Cohorts in the ELAPSE Project. Research Reports: Health Effects Institute 208. Health Effects Institute, Boston, MA PMC9476567 36106702

[CR10] Chen D, Zhang F, Yu C, Jiao A, Xiang Q, Yu Y, Mayvaneh F, Hu K, Ding Z, Zhang Y (2019). Hourly associations between exposure to ambient particulate matter and emergency department visits in an urban population of Shenzhen, China. Atmos Environ.

[CR11] Connell Hatch (2008) Final Report Environmental Evaluation of Fugitive Coal Dust Emissions from Coal Trains Goonyella, Blackwater and Moura Coal Rail Systems Queensland Rail Limited Report no. H327578-N00-EE00.00. (March 31, 2008). https://majorprojects.planningportal.nsw.gov.au/prweb/PRRestService/mp/01/getContent?AttachRef=MP09_0024%2120190814T054754.584%20GMT

[CR12] Dubovik O, Holben B, Eck TF, Smirnov A, Kaufman YJ, King MD, Tanre D, Slutsker I (2002). Variability of Absorption and Optical Properties of Key Aerosol Types Observed in Worldwide Locations. J Atmos Sci.

[CR13] DSITIA (2015) Initial Report on the Independent Review of Rail Coal Dust Emissions Management Practices in the NSW Coal Chain. https://www.chiefscientist.nsw.gov.au/__data/assets/pdf_file/0009/79884/Initial-Report_Review-rail-coal-dust-emissions.pdf. Accessed 15 Oct 2022

[CR14] Fuller R, Landrigan PJ, Balakrishnan K, Bathan G, Bose-O'Reilly S, Brauer M, Caravanos J, Chiles T, Cohen A, Corra L, Cropper M, Ferraro G, Hanna J, Hanrahan D, Hu H Hunter D, Janata G, Kupka R, Lanphear B, ... Yan C (2022) Pollution and health: a progress update. Lancet Planet Health 6(6):e535-e547. 10.1016/S2542-5196(22)00090-010.1016/S2542-5196(22)00090-035594895

[CR15] Hatch J, Affolter R, Davis F (1979). Chemical analyses of coal from the Blackhawk Formation, Wasatch Plateau coal field, Carbon, Emery, and Sevier Counties, Utah. Utah Geological and Mineral Survey Coal Studies.

[CR16] Higginbotham N, Ewald B, Mozeley F, Whelan J (2013) Coal Train Signature Study, Briefing Paper Prepared for Coal Terminal Action Group Dust and Health Committee. Coal Terminal Action Group (August, 2013):pp1–26. https://cdn.newsnow.io/storypad-bzDwk8TrKQYKjEefACKcS8/CoalTrainSignatureReportAug2013.pdf

[CR17] Hricko A, Rowland G, Eckel S, Logan A, Taher M, Wilson J (2014). Global Trade, Local Impacts: Lessons from California on Health Impacts and Environmental Justice Concerns for Residents Living near Freight Rail Yards. Int J Environ Res Public Health.

[CR18] Jaffe D, Putz J, Hof G, Hof G, Hee J, Lommers-Johnson DA, Gabela F, Fry JL, Ayres B, Kelp M, Minsk M (2015). Diesel particulate matter and coal dust from trains in the Columbia River Gorge, Washington State, USA. Atmos Pollut Res.

[CR19] Jha A, Muller M (2017) Handle with Care: The Local Air Pollution Costs of Coal Storage. National Bureau of Economic Research Report no. w23417, Cambridge, MA. 10.3386/w23417

[CR20] Katestone Environmental Pty Ltd. (2013). Pollution Reduction Program 4.2 Particulate Emissions from Coal Trains. Queensland: Prepared for Australian Rail Track Corporation Pty Ltd.

[CR21] Kim J, Kim H, Kweon J (2015) Hourly differences in air pollution on the risk of asthma exacerbation. Environ Pollut (Barking, Essex: 1987) 203:15–21 10.1016/j.envpol.2015.03.040 10.1016/j.envpol.2015.03.040 25845357

[CR22] Liu L, Song F, Fang J, Wei J, Ho HC, Song Y, Zhang Y, Wang L, Yang Z, Hu C, Zhang Y (2021) Intraday effects of ambient PM1 on emergency department visits in Guangzhou, China: A case-crossover study. Sci Total Environ 750:142347 10.1016/j.scitotenv.2020.142347 10.1016/j.scitotenv.2020.142347 33182206

[CR23] McDuffie E, Martin R, Brauer M (2015) A Global Assessment of Burden of Disease from Exposure to Major Air Pollution Sources: Synopsis of Research Report 210. December. https://www.healtheffects.org/system/files/mcduffie-rr-210-statement.pdf. Accessed 22 Sept 2022

[CR24] Meyer, R. (2019). A Major but Little-Known Supporter of Climate Denial: Freight Railroads. *Atlantic*, 2019–12–13T17:43:47Z https://www.theatlantic.com/science/archive/2019/12/freight-railroads-funded-climate-denial-decades/603559/

[CR25] Mikati I, Benson AF, Luben TJ, Sacks JD, Richmond-Bryant J (2018). Disparities in Distribution of Particulate Matter Emission Sources by Race and Poverty Status. Am J Public Health.

[CR26] OEHHA (2015) Risk Assessment Guidelines: Guidance Manual for Preparation of Health Risk Assessments, Appendices A-N. Air Toxics Hot Spots Program, Office of Environmental Health Hazards Assessment. February 2015. https://oehha.ca.gov/media/downloads/crnr/2015guidancemanual.pdf

[CR27] Ostro B, Hu J, Goldberg D, Reynolds P, Hertz A, Bernstein L, Kleeman MJ (2015). Associations of Mortality with Long-Term Exposures to Fine and Ultrafine Particles, Species and Sources: Results from the California Teachers Study Cohort. Environ Health Perspect.

[CR28] Ouimette JR, Malm WC, Schichtel BA, Sheridan PJ, Andrews E, Ogren JA, Arnott WP (2022). Evaluating the PurpleAir monitor as an aerosol light scattering instrument. Atmos Meas Tech.

[CR29] Peters A, Dockery DW, Muller JE, Mittleman MA (2001). Increased particulate air pollution and the triggering of myocardial infarction. Circulation.

[CR30] Prakash BB, Kecojevic V, Lashgari A (2018). Analysis of dust emission at coal train loading facility. Int J Min Reclam Environ.

[CR31] Ryan L, Wand M (2014) Re-analysis of ARTC Data on Particulate Emissions from Coal Trains. NSW Environment Protection Authority. (February 25, 2014):1–24. https://www.epa.nsw.gov.au/~/media/EPA/Corporate%20Site/resources/air/ARTCreanalysisFeb2014.ashx

[CR32] Sahu SP, Pakra AK (2022). Assessment of dispersion of respirable particles emitted from opencast mining operations: development and validation of stepwise regression models. Environ Dev Sustain.

[CR33] Srivastava A, Kumar A, Elumalai SP (2021). Evaluating Dispersion Modeling of Inhalable Particulates (PM10) Emissions in Complex Terrain of Coal Mines. Environ Model Assess.

[CR34] Tessum CW, Paolella DA, Chambliss SE, Apte JS, Hill JD, Marshall JD (2021) PM_2.5_ polluters disproportionately and systemically affect people of color in the United States. Sci Adv 7(18): eabf4491 10.1126/sciadv.abf4491 10.1126/sciadv.abf4491 PMC11426197 33910895

[CR35] Trivedi R, Chakraborty MK, Tewary BK (2009). Dust dispersion modeling using fugitive dust model at an opencast coal project of Western Coalfields Limited, India. J Sci Ind Res.

[CR36] Tsai CJ, Nair U, Hafner H (Eds.) (2020) Low-cost Sensors for Air Quality Monitoring, Aerosol Air Qual Res 20(2). https://aaqr.org/articles/20/2. Accessed Jan 2022

[CR37] US Energy Information Administration (2022) Annual Energy Outlook. https://www.eia.gov/outlooks/aeo/

[CR38] U.S. EPA (2019) Integrated Science Assessment (ISA) for Particulate Matter (Final Report, Dec 2019). U.S. Environmental Protection Agency, Washington, DC. https://cfpub.epa.gov/ncea/isa/recordisplay.cfm?deid=347534 36630543

[CR39] Vohra K, Vodonos A, Schwartz J, Marais EA, Sulprizio M, Mickley LJ (2021) Global mortality from outdoor fine particle pollution generated by fossil fuel combustion: Results from GEOS-Chem. Environ Res 195:110754. 10.1016/j.envres.2021.110754 10.1016/j.envres.2021.110754 33577774

[CR40] WHO (2021) WHO global air quality guidelines: Particulate matter (PM_2.5_ and PM10), ozone, nitrogen dioxide, sulfur dioxide and carbon monoxide (License: CC BY-NC-SA 3.0 IGO; pp. xxi, 273). World Health Organization. https://apps.who.int/iris/handle/10665/345329 34662007

[CR41] Wu PC, Cheng TJ, Kuo C-P, Fu, JS, Lai, H-C, Chiu T-Y, Lai, L-W (2020) Transient risk of ambient fine particulate matter on hourly cardiovascular events in Tainan City, Taiwan. PLoS One 15(8):e0238082. 10.1371/journal.pone.0238082 10.1371/journal.pone.0238082 PMC7442245 32822436

[CR42] Yorifuji T, Suzuki E, Kashima S (2014). Cardiovascular Emergency Hospital Visits and Hourly Changes in Air Pollution. Stroke.

